# Dyserythropoietic anaemia with an intronic *GATA1* splicing mutation in patients suspected to have Diamond‐Blackfan anaemia

**DOI:** 10.1002/jha2.374

**Published:** 2022-01-10

**Authors:** Akie Kobayashi, Ryusei Ohtaka, Tsutomu Toki, Junichi Hara, Hideki Muramatsu, Rika Kanezaki, Yuka Takahashi, Tomohiko Sato, Takuya Kamio, Ko Kudo, Shinya Sasaki, Taro Yoshida, Taiju Utsugisawa, Hitoshi Kanno, Kenichi Yoshida, Yasuhito Nannya, Yoshiyuki Takahashi, Seiji Kojima, Satoru Miyano, Seishi Ogawa, Kiminori Terui, Etsuro Ito

**Affiliations:** ^1^ Department of Pediatrics Hirosaki University Graduate School of Medicine Hirosaki Japan; ^2^ Department of Pediatric Hematology and Oncology Osaka City General Hospital Osaka Japan; ^3^ Department of Pediatrics Nagoya University Graduate School of Medicine Nagoya Japan; ^4^ Department of Transfusion Medicine and Cell Processing Faculty of Medicine Tokyo Women's Medical University Tokyo Japan; ^5^ Department of Pathology and Tumor Biology Graduate School of Medicine Kyoto University Kyoto Japan; ^6^ Division of Hematopoietic Disease Control Institute of Medical Science The University of Tokyo Tokyo Japan; ^7^ M&D Data Science Center Tokyo Medical and Dental University Tokyo Japan; ^8^ Department of Medicine Center for Hematology and Regenerative Medicine Karolinska Institute Stockholm Sweden; ^9^ Department of Community Medicine Hirosaki University Graduate School of Medicine Hirosaki Japan

**Keywords:** Diamond‐Blackfan anaemia (DBA), dyserythropoietic anaemia, GATA1, inherited bone marrow failure syndrome (IBMFS), intronic mutation

## Abstract

Diamond‐Blackfan anaemia (DBA) shares clinical features with two recently reported sporadic cases of dyserythropoietic anaemia with a cryptic *GATA1* splicing mutation (c.871‐24 C>T). We hypothesized that some patients clinically diagnosed with DBA but whose causative genes were unknown may carry the intronic *GATA1* mutation. Here, we examined 79 patients in our DBA cohort, who had no detectable causative genes. The intronic *GATA1* mutation was identified in two male patients sharing the same pedigree that included multiple cases with anaemia. Cosegregation of this mutation and disease in multiple family members provide evidence to support the pathogenicity of the intronic *GATA1* mutation.

## 1 INTRODUCTION

Diamond‐Blackfan anaemia (DBA) is one of the inherited bone marrow failure syndromes (IBMFSs), characterized by macrocytic pure red cell aplasia, variable malformations and a predisposition to malignancies [1]. It generally presents in the first year of life. DBA patients generally exhibit increased levels of foetal haemoglobin (HbF). The activity of erythrocyte adenosine deaminase (eADA) and the concentration of erythrocyte reduced glutathione (GSH) are elevated in 75%–90% and 59.1% of DBA patients, respectively [1, 2, 3]. Nearly 60% of DBA patients will respond to steroids over the long term [1]. To date, 22 ribosomal protein (RP) genes have been reported as causative genes of DBA in close to 70% of patients [1, 4, 5, 6, 7]. All known DBA‐causative mutations involve RP genes, except for rare germline *GATA1* and *TSR2* mutations [8, 9]. Missense mutations in *GATA1* cause DBA, congenital dyserythropoietic anaemia, thalassemia and a variety of thrombopoietic defects [10]. Recently, Abdulhay et al. reported two sporadic dyserythropoietic anaemia cases with an intronic mutation in *GATA1* (c.871‐24 C>T). This mutation results in reduced canonical splicing and increased use of an alternative splice acceptor site that causes a partial intron retention event, leading to a five amino acids insertion at the C‐terminal zinc finger domain of *GATA1* [11]. The resultant altered GATA1 protein has no observable activity. These cases and DBA patients share common clinical features including the early onset of severe macrocytic anaemia with an inadequate reticulocyte response, elevated HbF and high eADA levels. Therefore, we hypothesized that some patients with a clinical diagnosis of DBA whose causative genes were unknown may harbour the intronic mutated *GATA1*.

## 2 RESULTS AND DISCUSSIONS

**FIGURE 1:**
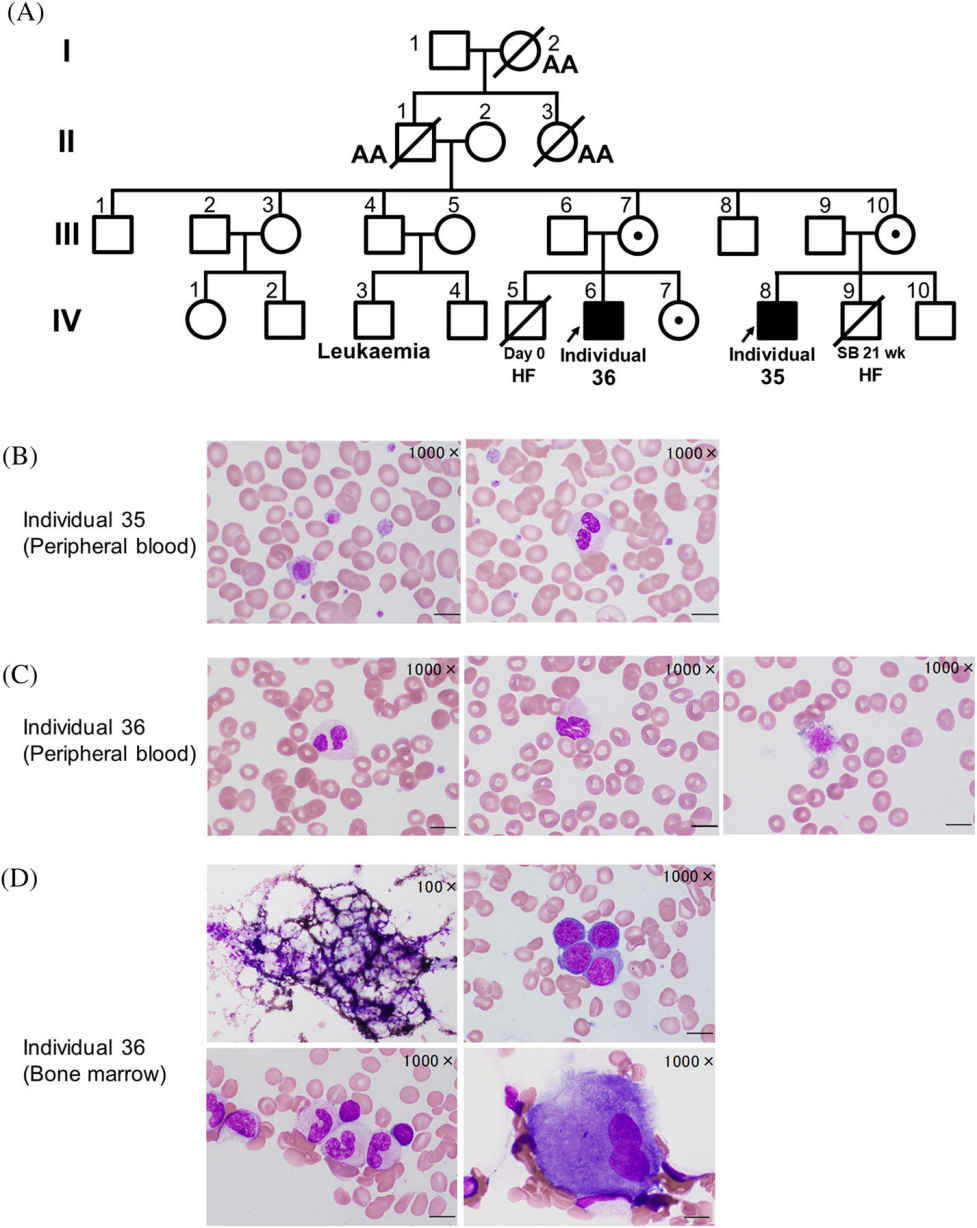
Dyserythropoietic anaemia with an intronic *GATA1* splicing mutation. (A) Pedigree of a family affected by a *GATA1* c.871‐24 C>T mutation. AA, aplastic anaemia; HF, hydrops foetalis; SB, stillbirth. We examined the intronic *GATA1* mutation by direct sequencing analysis of 79 patients in our Diamond‐Blackfan anaemia (DBA) cohort, who had no detectable causative genes for DBA by target sequencing and whole‐exome sequencing analysis. (B) Peripheral blood smear images (May–Giemsa stain) from individual 35 show large platelets (left) and neutrophil dysplasia like pseudo‐Pelger anomaly (right). (C) Peripheral blood smear images from individual 36 show neutrophil dysplasia like pseudo‐Pelger anomaly (left), degranulated neutrophil (middle) and a giant platelet (right). (D) Bone marrow smear images (May–Giemsa stain) from individual 36 show hypocellularity (left upper) with trilineage dysplasia, hypo‐segmented mature neutrophils (left lower), megaloblastoid changes (right upper) and hypo‐segmented megakaryocytes (right lower). (B to D): Scale bars: 10 μm

Our DBA cohort consisted of 215 individuals, including 109 males. Among them, we examined the intronic *GATA1* mutation by direct sequencing analysis in 79 individuals (including 47 males), for whom the causative gene could not be identified by target and whole‐exome sequencing (Table S1). All clinical samples were obtained with informed consent from the patients and their families. The ethics committee of Hirosaki University Graduate School of Medicine approved this study. Using their genomic deoxyribonucleic acid (DNA) samples, we performed mutation analyses of the region containing chrX:48,652,176 in hg19; *GATA1* c.871‐24 by polymerase chain reaction (PCR) and Sanger sequencing analysis. We detected the *GATA1* c.871‐24 C>T mutation in a family in which multiple individuals with anaemia were observed. This family included two male patients (individuals 35 and 36) (Figure 1A).

Individual 35 was born at term after an uncomplicated pregnancy. He had a ventricular septal defect. His younger brother was diagnosed with hydrops foetalis and died at the gestational age of week 21. At the age of 1 year, individual 35 visited a hospital because of anaemia. Peripheral blood (PB) analysis showed Hb 7.0 g/dl, mean corpuscular volume (MCV) 99.6 femto Liter (fL), reticulocytes 0/μl, white blood cells 10,100/μl and platelets 287,000/μl and BM aspiration showed hypocellularity and erythroid hypoplasia. A probable diagnosis of DBA was considered, and corticosteroid therapy was initiated. He showed clinical responses to steroid therapy, and cyclosporine was added to reduce the dosage of corticosteroid. However, following cyclosporine combination therapy was abandoned due to renal dysfunction. Since then, he has been on low‐dose steroid therapy without blood transfusion. His platelet counts remained in the normal range. However, there were large platelets on the PB smear (Figure 1B), and his platelet adhesion assay level was as low as 8.9% (normal control: 15%–45%). In addition, the smear showed neutrophil dysplasia (Figure 1B). At the age of 15 years, his eADA and GSH levels were 1.09 (normal 0.46–1.50) IU/gHb and 124 (normal 62.4–98.9) mg/dl red blood cell (RBC), respectively, suggesting a DBA pattern using a previously reported formula [2].

Individual 36 was born at term. He had hypospadias and cryptorchidism. His older brother was diagnosed with hydrops foetalis and died 9 h after birth (Figure 1A). Individual 36 had been under observation for mild anaemia since his neonatal period. At the age of 3 years, PB analysis showed anaemia with macrocytosis (Hb 8.2 g/dl, MCV 108.4 fL, reticulocytes 38,080/μl) and mild thrombocytopenia (platelets 139,000/μl). His HbF was elevated to 4.4%. DBA was considered the probable diagnosis, and he was treated with a combination therapy consisting of corticosteroid and cyclosporine with good clinical responses. From the age of 9, he had been followed without medication or blood transfusion. However, at the age of 14 years, influenza virus infection occurred with worsened anaemia. Since the anaemia persisted after that, the combination therapy was restarted, but anaemia improved insufficiently. On the PB smear, multiple lineage dysplasia was observed (Figure 1C). BM examinations performed at the age of 15 years showed hypocellularity with trilineage dysplasia (Figure 1D). His condition was complicated by Behcet's disease, and he had lower gastrointestinal bleeding from an ileocecal ulcer. Furthermore, he developed haemophagocytic lymphohistiocytosis and received immunosuppressive therapy with dexamethasone and cyclosporine. However, he did not recover from the BM failure and underwent an allogeneic BM transplantation from an human leukocyte antigen (HLA)‐matched unrelated donor. Engraftment of neutrophils was confirmed 28 days after transplantation, but haematopoiesis was still insufficient, and transfusion of red blood cells and platelets was required.

The two individuals with the intronic *GATA1* mutation in our DBA cohort had clinical phenotypes similar to the two individuals reported by Abdulhay et al. [11]. They shared several characteristics, including the early onset of macrocytic anaemia, elevated HbF, mild trilineage dysplasia, abnormal platelet function and exacerbation of anaemia upon infection (Table S2). In both of our cases, their siblings died of hydrops foetalis, and one of the previously reported cases also received intrauterine transfusion for hydrops foetalis [11], suggesting that male individuals with this mutation have a higher incidence of hydrops foetalis and should be given more attention in perinatal management. However, there are some differences between our cases and the individuals from the previous report. For example, in our cases, anaemia improved in response to corticosteroids, whereas the previous cases recovered spontaneously from severe anaemia without medication. Although the mechanism of steroid effect is unknown, steroid therapy seems to be a treatment worth trying. In contrast to the previous cases, one of our cases had a prolonged BM failure triggered by an infection, which required an allogeneic BM transplantation. Even after the spontaneous remission of this disease, an infection could lead to BM failure.

**FIGURE 2:**
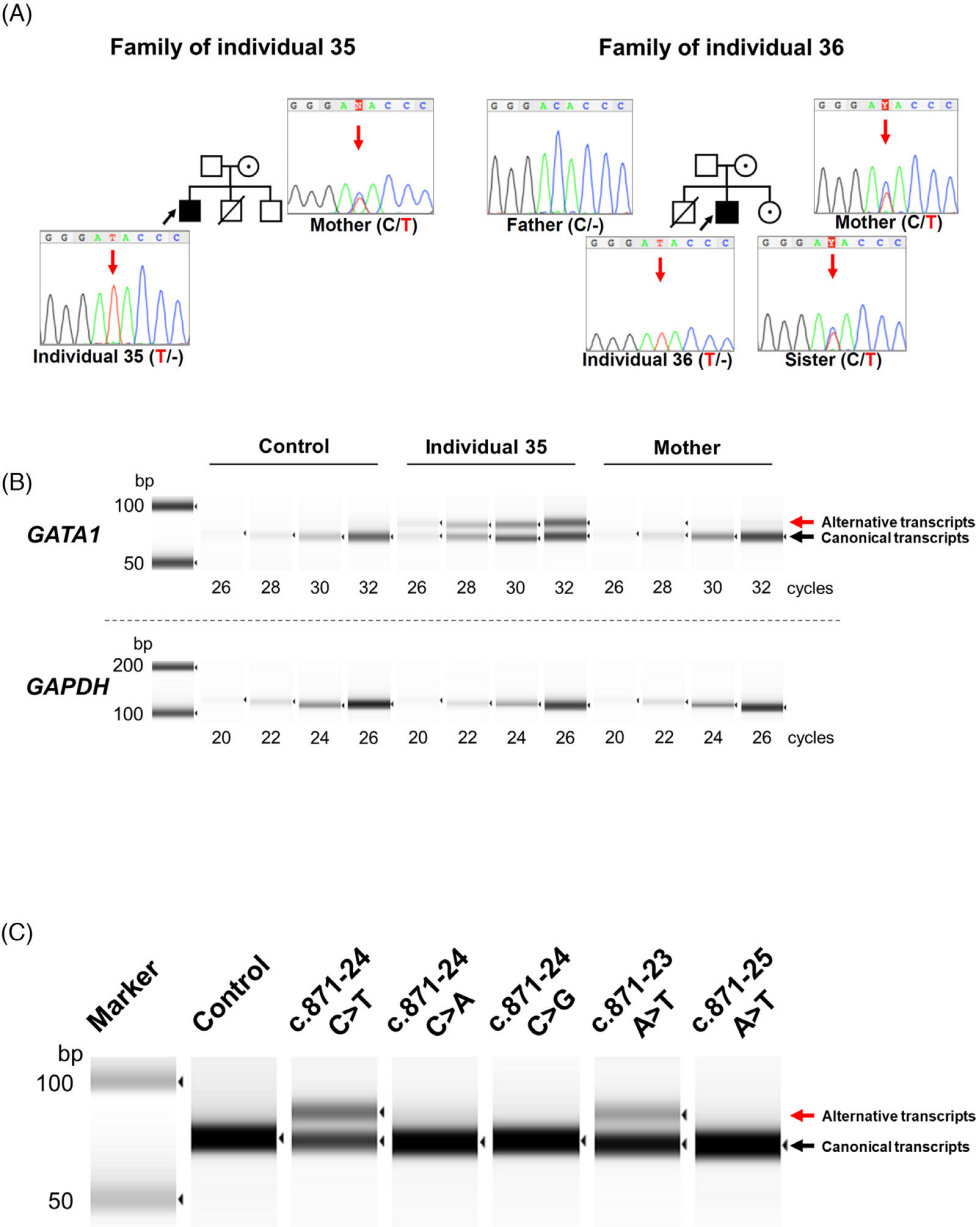
Identification of intronic *GATA1* mutations. (A) Sanger sequencing chromatograms of *GATA1* intron 5. Red arrows indicate the position of nucleotide substitution c. 871‐24 C>T. (B) Semiquantitative RT‐PCR analysis of *GATA1* exons 5 and 6 using total RNA purified from peripheral blood of individual 35, his mother and a healthy control. Abundant alternative messenger RNA (mRNA) with intron retention was detected in addition to wild‐type mRNA in individual 35. (C) RT‐PCR analyses were performed using BHK‐21 cells expressing several *GATA1* mutations by the minigene technique. *GATA1* c.871‐24 C>T and c.871‐23 A>T led to expression of an identical alternative transcript

Notably, the pedigree included three older members (I‐2, II‐1, 3) who had been diagnosed with aplastic anaemia (Figure 1A). We analysed the genotype of the mutant allele of four family members whose samples were available (mother of individual 35: III‐10, and parents and sister of individual 36: III‐6, 7, IV‐7). Three female members had the *GATA1* mutation heterozygously, and the remaining male member (father of individual 36: III‐6) had a wild‐type allele hemizygously (Figure 2A). To confirm the effect of the mutation, we performed semiquantitative reverse transcription PCR (RT‐PCR) analyses using total ribonucleic acid (RNA) purified from the PB of individual 35 and his mother as well as a healthy control, using previously reported primers (Figure 2B) [11]. We found a longer *GATA1* transcript that retained the 15 nucleotides of the 5th intron by alternative splicing, in addition to the canonical transcript in individual 35, whereas a trace amount was found in the healthy control. DNA quantitation using TapeStation system (Agilent) showed the percentages of the canonical transcripts of total *GATA1* in individual 35 and the healthy control were 57% and almost 100%, respectively, suggesting a reduction of normal transcript in individual 35 compared to healthy control (Figure 2B). In individual 35 with the hemizygous *GATA1* mutation, the alternative transcripts constituted 43% of the total *GATA1* transcripts. Given that the X‐chromosome inactivation is a random process, the percentage of altered transcripts should have been about 20% in the heterozygous mother. However, the actual value was as low as 2.7%. This result may suggest that clones expressing the mutant allele are suppressed in the maternal haematopoietic system.

It is difficult to predict which intronic base substitutions will cause splicing abnormalities. We investigated the possibility that the mutations around the substitution sites found in this study could cause similar splicing abnormalities using splice site prediction software, NetGene2 [12, 13]. We found that c.871‐24 C>T increased the acceptor prediction score from 0.054 to 0.067. However, there was no change in either c.871‐24 C>A or C>G. When various mutations were entered, the expected score was shown to increase to 0.1 for c.871‐23 A>T and c.871‐25 A>T (Table S3). Because this change was larger than for c.871‐24 C>T, RT‐PCR analysis was performed with these mutations in the minigene construct as described previously [14]. As expected, c.871‐24 C>A and C>G did not cause splicing abnormalities, and c.871‐23 A>T showed splicing abnormalities with slightly lower efficiency than c.871‐24 C>T. However, c.871‐25 A>T showed no abnormal transcripts, contrary to our expectation. These results indicate that similar splicing abnormalities may be caused by c.871‐23 A>T (Figure 2C).

Interestingly, there are three older members with aplastic anaemia in this pedigree. Although we could not obtain samples for further study, they may have heterozygous or hemizygous intronic *GATA1* mutation (Figure 1A). It is known that IBMFSs patients develop aplastic anaemia and have a predisposition for malignancies; including myelodysplastic syndrome (MDS) or acute myeloid leukaemia [15]. This study indicates that even if an individual carries this *GATA1* mutation heterozygously, the possibility of developing aplastic anaemia or MDS at an older age might not be excluded.

In conclusion, we examined the intronic *GATA1* mutation by direct sequencing analysis of 79 patients without known DBA causative genes in our DBA cohort. We detected the intronic *GATA1* mutation in two male patients in a family with multiple individuals affected by anaemia, sharing many clinical characteristics with two individuals from a previous report [11]. Cosegregation of this mutation and disease in multiple family members provided supporting evidence of the pathogenicity. These results further support the concept that dyserythropoietic anaemia with the intronic *GATA1* mutation is a distinct clinical entity and demonstrate the usefulness of screening for the intronic *GATA1* mutation in the diagnostic evaluation of congenital anaemia.

## AUTHOR CONTRIBUTIONS

Akie Kobayashi, Ryusei Ohtaka, Tsutomu Toki, Rika Kanezaki, Yuka Takahashi, Taiju Utsugisawa, Hitoshi Kanno and Kenichi Yoshida performed research. Akie Kobayashi, Tsutomu Toki and Etsuro Ito wrote the manuscript. Kenichi Yoshida, Yasuhito Nannya and Satoru Miyano performed bioinformatics analyses of the resequencing data. Junichi Hara, Hideki Muramatsu, Taro Yoshida, Yoshiyuki Takahashi, Seiji Kojima, Tomohiko Sato, Takuya Kamio, Ko Kudo and Shinya Sasaki collected specimens and clinical data. Etsuro Ito, Tsutomu Toki, Kiminori Terui and Seishi Ogawa were involved in planning the project.

## CONFLICTS OF INTEREST

The authors declare no conflict of interest.

## Supporting information

Supporting Information
