# Supplementary material for: Dyserythropoietic anaemia with an intronic GATA1 splicing mutation in patients suspected to have Diamond‐Blackfan anaemia
Source: EJHaem. 2022 Jan 10;3(1):163–7. doi: 10.1002/jha2.374 (PMC9175706; doi:10.1002/jha2.374)
Supplement: Supplementary file 1 — Supporting Information [file JHA2-3-163-s001.pdf]

Supplementary Table S1. Characteristics of 79 patients in our DBA cohort subjected to target sequencing

Patient (UPN)	Age at diagnosis	Gender	Inheritance	WBC (/μL)	Hb (g/dL)	MCV (fL)	Plt (x10e3/μL)	Abnormality	Mutation	Response to first steroid therapy
**5**	1 year	F	Sporadic	27,400	3.1	NA	5490	None	ND	Yes
**7**	1 month	M	Sporadic	7,600	5.6	92.1	79	Skin pigmentation, low-set ears, hypertelorism, growth retardation, single transverse palmar crease and SFD	ND	Yes
**13**	3 months	F	Sporadic	14,800	2.5	97.6	839	None	ND	No
**26**	13 months	F	Sporadic	8,700	7.3	106	668	Congenital hip dysplasia, spastic quadriplegia, intense myopia, optic nerve hypopasia, learning disabilities and growth retardation	ND	No
**33**	2 months	F	Sporadic	14,500	1.3	137	91	Growth retardation and learning disabilities	ND	Yes
**35**	18 months	M	Familial	10,100	7.0	99.6	287	Congenital heart disease	*GATA1* c.871-24 C>T	Yes
**36** (Cousin of UPN 35)	Neonatal period	M	Familial	7,800	8.2	108.4	139	Hypospadias, cryptorchism, Behcet disease, pervasive developmental disorder, precocious puberty, lower gastrointestinal bleeding and HLH	*GATA1* c.871-24 C>T	Yes
**37**	4 years	M	Sporadic	6,500	6.1	84.1	646	Hypospadias and cryptorchism	ND	NT
**49**	2 months	M	Sporadic	5,500	2.0	105.0	324	Growth retardation and SFD	ND	Yes
**50**	16 months	F	Familial	4,000	3.4	87.7	285	Growth retardation	ND	Yes
**51**	6 months	F	Sporadic	9,000	4.2	70.2	481	LFD	ND	No
**52** (Sister of UPN 50)	6 months	F	Familial	7,300	6.8	79.4	307	None	ND	Yes
**53**	9 months	F	Sporadic	5,900	2.2	74.8	403	SFD	ND	NT
**54**	8 years	F	Sporadic	4,220	5.3	87.4	308	None	ND	NT
**61**	9 months	M	Sporadic	15,040	4.0	77.9	479	None	ND	Yes
**67**	3 years	M	Sporadic	5,400	6.2	77.2	276	None	ND	NT
**68**	16 months	M	Sporadic	4,800	5.9	96.3	155	None	ND	NT
**69**	18 months	M	Sporadic	9,780	8	88.8	561	None	ND	Yes
**77**	17 days	M	Familial	9,250	7.8	106.0	1,008	Growth retardation	ND	No
**83**	10 months	M	Sporadic	7,900	3.0	76.0	188	None	ND	No
**93**	14 months	M	Sporadic	21,090	2.2	122.4	224	Syndactyly, hypodactyly, corneal opacity, growth retardation, sparse hair, sparse eyeblow, leukoderma, lymphedema and cryptorchism	ND	Yes
**96**	2 years	F	Sporadic	11,000	1.9	84.6	559	None	ND	No
**97**	4 years	F	Sporadic	5,900	8.8	99.2	407	Growth hormone deficiency and SFD	ND	Yes
**105**	1 month	M	Sporadic	3,800	5.1	100.0	352	Short stature	ND	No
**112**	4 months	F	Sporadic	6,800	8.5	89.9	155	Polydactyly thumb, low-set ears, high nasal bridge, cerebellar hypoplasia, congenital heart disease and growth retardation	ND	NT
**116**	4 months	M	Sporadic	12,400	0.3	100.0	780	Flat thenar eminence	ND	NT
**135**	13 months	M	Sporadic	34,350	3.6	72.5	373	Xanthogranuloma and inguinal hernia	ND	NA
**136**	1 month	M	Sporadic	6,570	3.5	115.0	554	None	ND	Yes
**151**	15 months	M	Sporadic	5,600	3.2	63.9	355	None	ND	Yes
**153**	16 months	M	Sporadic	5,000	2.1	75.6	379	Growth retardation	ND	No
**158**	5 months	M	Sporadic	9,600	4.8	91.1	704	Congenital heart disease	ND	NT
**159**	11 months	M	Sporadic	8,600	5.9	84.4	262	Growth retardation	ND	Yes
**219**	Neonatal period	F	Sporadic	8,780	4.5	129.5	5	SGA, growth retardation, tracheomalacia and subglottic stenosis	ND	NT
**233**	21 months	F	Sporadic	9,100	4.4	81.0	601	None	ND	No
**236**	3 years	M	Sporadic	5,300	2.1	81.8	485	Myocardial hypertrophy	ND	NT
**247**	10 months	M	Sporadic	10,200	6.7	103.0	183	None	ND	Yes
**250**	Neonatal period	M	Sporadic	10,600	2.6	NA	62	Congenital aural atresia, accessory auricle, hypoplastic kidney, hypospadias, growth retardation and single transverse palmar crease	ND	NT
**251**	2 months	F	Sporadic	6,200	5.7	85.9	540	None	ND	Yes
**252**	3 months	F	Sporadic	8,000	8.3	89.5	232	Hydrocephalus	ND	NT
**253**	23 months	M	Sporadic	6,300	2.1	72.4	416	None	ND	Yes
**264**	18 months	M	Sporadic	NA	3.0	NA	NA	None	ND	No
**269**	18 days	F	Sporadic	9,260	3.4	112.0	977	NA	ND	NT
**273**	5 months	M	Sporadic	6,600	2.2	88.4	394	NA	ND	No
**281**	11 months	M	Sporadic	9,500	4.4	84.4	434	None	ND	NT
**285**	1 month	F	Sporadic	7,070	4.8	85.3	303	None	ND	NT
**290**	14 months	M	Sporadic	10,230	6.7	115.3	398	Funnel chest	ND	NT
**303**	9 months	M	Sporadic	6,400	2.2	84.0	338	Congenital heart disease	ND	NT
**304**	7 months	F	Sporadic	13,100	3.3	87.2	631	None	ND	NT
**307**	7 months	M	Sporadic	4,950	6.9	80.6	173	Growth retardation	ND	NA
**312**	4 months	F	Sporadic	13,900	3.3	78.9	467	None	ND	NA
**315**	16 months	M	Sporadic	6,900	2.3	75.3	421	None	ND	NT
**318**	6 months	M	Sporadic	14,700	9.3	82.5	158	Congenital heart disease and growth retardation	ND	NA
**329**	1 months	F	Sporadic	7,730	9.1	97.5	NA	None	ND	NT
**333**	4 months	M	Sporadic	9,500	5.1	94.2	822	None	ND	NA
**341**	10 months	F	Sporadic	8,600	2.8	81.0	493	None	ND	Yes
**343**	47 years	F	Familial	6,100	7.1	115.0	215	Breast cancer	ND	NT
**346**	7 months	F	Sporadic	7,200	3.3	99.3	599	Congenital heart disease	ND	Yes
**350**	15 montha	F	Sporadic	8,000	6.1	83.6	274	Growth retardation	ND	NT
**353**	12 months	M	Sporadic	12,490	3.5	84.1	550	None	ND	NT
**360**	4 months	M	Sporadic	9,850	8.1	98.0	472	Inguinal hernia	ND	NT

**362**	2 months	M	Sporadic	8,200	6.8	89.6	229	Growth retardation and SGA	ND	NT
**368**	3 months	M	Sporadic	7,240	6.2	96.6	215	Short stature and liver dysfunction	ND	NT
**379**	11 months	F	Sporadic	11,200	5.5	102.3	708	SGA and precocious puberty	ND	NA
**380**	1 month	M	Sporadic	6,100	5.8	90.7	177	Low-set ears and micrognathia	ND	NT
**387**	9 years	F	Sporadic	4,200	5.8	81.2	309	None	ND	NT
**389**	13 months	M	Sporadic	7,200	2.6	82.0	364	None	ND	NT
**395**	1 month	F	Familial	6,700	7.5	106.8	466	None	ND	NT
**396** (Mother of UPN 395)	37 years	F	Familial	7,000	11.9	116.3	98	None	ND	NT
**397**	11 months	M	Sporadic	11,960	10.9	81.0	164	Dilated cardiomyopathy	ND	NT
**398**	2 months	F	Sporadic	6,000	2.9	92.5	405	None	ND	NT
**401**	9 years	M	Sporadic	3,400	8.0	86.0	330	Congenital heart disease	ND	No
**406**	15 months	F	Sporadic	8,500	8.8	85.5	291	Congenital heart disease	ND	NT
**407**	16 months	M	Sporadic	4,800	4.3	81.9	251	None	ND	NT
**413**	11 years	M	Sporadic	6,710	5.5	94.6	386	None	ND	No
**416**	11 months	F	Sporadic	8,900	4.7	76.5	354	None	ND	NT
**421**	1 month	M	Sporadic	11,870	7.8	89.5	273	SGA	ND	NT
**430**	11 months	M	Sporadic	21,500	3.1	78	668	None	ND	Yes
**434**	17 months	M	Sporadic	9,000	5.7	80.9	492	None	ND	NT
**435**	14 months	M	Sporadic	9,580	6.6	89.9	349	Growth retardation and retinal choroidal atrophy	ND	NT

UPN: unique patient number; SFD: small for dates; LFD: light for dates; HLH: Hemophagocytic lymphohistiocytosis; SGA: small for gestational age; NA: not available; ND: not detected; NT: not tested

Supplementary Table S2. Clinical characteristics of the individuals carrying an intronic mutation in *GATA1* (c.871-24 C>T)

Case	Individual 35	Individual 36	Individual 1*	Individual 2*
**Gender**	M	M	M	M
**Gestational age**	Term	Term	Term	Term
**Age at diagnosis**	1 year	Neonatal period	1 day	Foetal period
**Hb (g/dL)**	7.0	8.2	9.4	2.4
**MCV (fL)**	99.6	108.4	Raised (-100)	Raised (-100)
**HbF (%)**	ND	4.4	20-23	6.5
**eADA (IU/gHb)**	Normal (1.09)	ND	Elevated (2.79)	Elevated (123)
**GSH (mg/dL RBC)**	Elevated (124)	ND	ND	ND
**Platelet dysfunction**	Yes	ND	Yes	Yes
**PB smear**	Pseudo-Pelger anomaly and large platelets	Pseudo-Pelger anomaly, hypo-segmented mature neutrophils and giant platelets	Giant platelets	Large platelets
**BM smear**	Hypocellularity and erythroid hypoplasia<br>(Original bone marrow smear samples were not available for reevaluation.)	Hypocellularity with trilineage dysplasia, hypo-segmented mature neutrophils, megaloblastoid changes and hypo-segmented megakaryocytes	Normocellularity with trilineage dysplasia, megaloblastoid changes, hypo-segemented megakaryocytes and degranulated neutrophils	Normocellularity with trilineage dysplasia, signs of dyserythropoiesis and small dysplastic megakaryocytes
**Exacerbation of anaemia upon infection**	No	Yes	Yes	Yes
**Complications**	Ventricular septal defect	Hypospadias, cryptorchism, Behcet disease, pervasive developmental disorder, precocious puberty, lower gastrointestinal bleeding and HLH	Hypospadias and lower gastrointestinal bleeding	No
**Family history**	Maternal great-grandmother, grandaunt and grandfather: AA<br>Maternal cousin #1: leukaemia<br>Maternal cousin #2 (Individual 36): DBA-like disease<br>Younger brother: Stillbirth due to hydrops foetalis	Maternal great-grandmother, grandaunt and grandfather: AA<br>Maternal cousin #1: leukaemia<br>Maternal cousin #2(Individual 35): DBA-like disease<br>Older brother: perinatal death due to hydrops foetalis	Maternal cousin: chronic anaemia	Maternal cousin: thalassemia

PB: peripheral blood; BM: bone marrow; ND: not done; AA: aplastic anaemia; DBA: Diamond Blackfan anaemia; HLH: Hemophagocytic lymphohistiocytosis. *Abdulhay NJ *et al*. J ExpMed 2019;216(5):1050-1060.

Supplementary Table S3 : Confidence of the splice-acceptor site prediction

Position		Wild		-24C>T		-23A>T		-25A>T		-24C>A		-24C>G	
	-32	G	0	G	0	G	0	G	0	G	0	G	0
	-31	T	0	T	0	T	0	T	0	T	0	T	0
	-30	T	0	T	0	T	0	T	0	T	0	T	0
	-29	G	0	G	0	G	0	G	0	G	0	G	0
	-28	G	0	G	0	G	0	G	0	G	0	G	0
	-27	G	0	G	0	G	0	G	0	G	0	G	0
	-26	G	0	G	0	G	0	G	0	G	0	G	0
	-25	A	0	A	0	A	0	T	0	A	0	A	0
	-24	C	0	T	0	C	0	C	0	A	0	G	0.054
	-23	A	0	A	0	T	0	A	0	A	0	A	0
	-22	C	0	C	0	C	0	C	0	C	0	C	0
	-21	C	0	C	0	C	0	C	0	C	0	C	0
	-20	C	0	C	0	C	0	C	0	C	0	C	0
	-19	G	0	G	0	G	0	G	0	G	0	G	0
	-18	C	0	C	0	C	0	C	0	C	0	C	0
	-17	A	0	A	0	A	0	A	0	A	0	A	0
	-16	G	0.054	G	0.067	G	0.100	G	0.100	G	0.054	G	0.054
	-15	C	0	C	0	C	0	C	0	C	0	C	0
	-14	C	0	C	0	C	0	C	0	C	0	C	0
	-13	T	0	T	0	T	0	T	0	T	0	T	0
	-12	C	0	C	0	C	0	C	0	C	0	C	0
	-11	C	0	C	0	C	0	C	0	C	0	C	0
	-10	T	0	T	0	T	0	T	0	T	0	T	0
	-9	T	0	T	0	T	0	T	0	T	0	T	0
	-8	T	0	T	0	T	0	T	0	T	0	T	0
	-7	T	0	T	0	T	0	T	0	T	0	T	0
	-6	T	0	T	0	T	0	T	0	T	0	T	0
	-5	G	0	G	0	G	0	G	0	G	0	G	0
	-4	G	0	G	0	G	0	G	0	G	0	G	0
	-3	C	0	C	0	C	0	C	0	C	0	C	0
	-2	A	0	A	0	A	0	A	0	A	0	A	0
**IVS 5**	-1	G	0.846	G	0.897	G	0.846	G	0.887	G	0.846	G	0.933
**Exon 6**	c.871	G	0	G	0	G	0	G	0	G	0	G	0
	c.872	T	0	T	0	T	0	T	0	T	0	T	0
	c.873	G	0	G	0	G	0	G	0	G	0	G	0
	c.874	A	0	A	0	A	0	A	0	A	0	A	0
	c.875	A	0	A	0	A	0	A	0	A	0	A	0
	c.876	C	0	C	0	C	0	C	0	C	0	C	0
	c.877	C	0	C	0	C	0	C	0	C	0	C	0
